# Modifying Graft-versus-Host Disease in a Humanized Mouse Model by Targeting Macrophages or B-Cells

**DOI:** 10.1155/2019/3538963

**Published:** 2019-05-08

**Authors:** Marieke C. H. Hogenes, Suzanne van Dorp, Joyce van Kuik, Filipa R. P. Monteiro, Natalie ter Hoeve, Liane Guedes, Marijke R. van Dijk, Anton C. Martens, Roel A. de Weger

**Affiliations:** ^1^Laboratory for Pathology East Netherlands, (LABPON), P.O. Box 510, 7550 AM Hengelo, Netherlands; ^2^Department of Pathology UMC Utrecht, Utrecht, Netherlands; ^3^Department of Hematology (DIGD) UMC Utrecht, Utrecht, Netherlands; ^4^Department of Hematology, VU University Medical Centre, Amsterdam, Netherlands

## Abstract

Humanized mouse models can well be modified to study specific aspects of Graft-versus-Host Disease (GvHD). This paper shows the results of both macrophage depletion and (early) B-cell depletion in a humanized mouse model using RAG2^−/−^*γ*c^−/−^ mice injected with HuPBMCs. Macrophage depletion showed a significant decrease in survival and also lead to a change in the histomorphology of the xenogeneic reaction. Higher levels of infiltrating B-cells were observed in various organs of mice depleted for macrophages. With (early) B-cell depletion using Rituximab, a clear improvement on clinical symptoms was observed, even when probably only inactivated B-cells were deleted. However, the histological examinations only showed a significant morphological effect on liver fibrosis. This may be related to a difference in the mRNA levels of TGF-*β*. Also, lower mRNA levels of Tregs in some organs were observed after Rituximab treatment, which contradicts that a higher number of Tregs would always be related to less severe GvHD. Our data show that both macrophage depletion and (early) B-cell depletion in a xenogeneic mouse model can influence the clinical, histological, and cytokine production of a GvHD response.

## 1. Introduction

Since researchers developed an interest in the pathogenesis and possible treatment targets of human Graft-versus-Host Disease (GvHD), the interest in mouse models has grown. Several mouse models, including humanized mouse models, are available as discussed in our previous paper [1] with endless options to modify the model to your needs. One of these models is the RAG2^−/−^*γ*c^−/−^ mouse model [1], in which the immunodeficient mice lack mature T-cells, mature B-cells, and NK-cells but maintain monocytes and macrophages.

The list of interesting research targets in GvHD is long and each step takes us closer to unraveling the complex reaction occurring after bone marrow transplant and donor lymphocyte infusion. In early days, one thought that cytotoxic T-cells were involved; today's knowledge shows an important role in GvHD for regulatory T-cells (Tregs) and B-cells as well. Tregs have a suppressive effect by downregulating the proliferative activity of T-cells [2]. For B-cells, a positive influence on GvHD activity has been noted when using Rituximab, a B-cell-depleting antibody [3–6]. Rituximab was originally developed to treat non-Hodgkin lymphomas [4, 7] but is successful in a variety of autoimmune diseases as well [8].

A possible interaction between Tregs and B-cells cannot be excluded. Previous research has shown an association of B-cell depletion with a change in the numbers of CD4+ and CD8+ T-cells, as well as changes in their activation status [9]. Early phase B-cell depletion is associated with prominent increased mRNA levels of genes characterized by Tregs in Rituximab-treated patients, including CD25, FoxP3, CTLA-4, GITR, and TGF-beta [10]. Rituximab treatment reduces CD4+ T-cells with the expression of the costimulatory/activation molecule CD40L [10]. Some studies suggested that the effect of B-cells on Tregs might be activation dependent [11–13]; others suggest that Tregs directly influence B-cells due to their presence not only in the paracortical zone but also in the mantle zone of a follicle [14].

We have noticed a difference in methods in both Tregs and B-cell studies, in which some researchers have used clodronate depletion to accompany the mouse conditioning regimen and some have not. Clodronate is a pharmakon associated with induction of apoptosis in monocytes, macrophages, and osteoclasts [15–19]. The half-life of clodronate is very short when released in circulation, and it does not easily cross cell membranes. However, to avoid this issue, one could embed clodronate in multilamellar liposomes, which are exclusively ingestible for phagocytic cells [20]. Once ingested, clodronate accumulates within macrophages, resulting in a toxic level of clodronate that ultimately leads to apoptosis of the macrophages [21]. After clodronate depletion therapy, the number and phenotype of the residual of any given tissue depend on the tissue being examined and will change during infection of inflammation [22]. Similarly, the ability to deplete macrophages using this protocol will depend on the route of administration. Intravenous injection, for example, results in depletion of macrophages from mainly the spleen and the liver within one day after single dose administration [20, 23]. Repopulation of these macrophages in the liver starts as soon as 5 days postinjection [24] and within a week in the spleen [25]. However, it cannot be excluded that donor macrophages might also be depleted with this protocol, as van Rijn et al. showed that donor CD14+ cells disappear soon after huPBMC injection when clodronate had been administered [26].

Even though it is known that different pre- and posttreatments in your humanized mouse model will have a direct effect on the severity of the displayed symptoms, less is known about the effects of depleting specific cell lines in the modification of a humanized mouse model that might influence the resulting type of reaction and its histomorphology or cytokine profile.

In this paper, we take a closer look on the effect of depleting host macrophages and donor B-cells on tissue morphology, as well as the resulting cytokine profile in the xenogeneic RAG2^−/−^*γ*c^−/−^ mice mouse model.

## 2. Materials and Methods

### 2.1. Mice and Conditioning Regimens

RAG2^−/−^*γ*c^−/−^ mice were obtained from the Netherlands Cancer Institute (Amsterdam, Netherlands) [27]. The mice were bred and maintained under microisolator cages under specified pathogen-free conditions at the Central Laboratory Animal Institute (Utrecht University, Netherlands) and were given sterile water and irradiated pellets ad libitum. All experiments were conducted after permission was granted by the local Ethical Committee for Animal Experiments and in accordance with the Dutch law on animal experimentation.

#### 2.1.1. Macrophage-Depletion Model

Macrophage depletion was studied in duplo, using the same conditions described by van Rijn et al. [26]. In both experiments, all mice were given total body irradiation (TBI) with a single dose of 350 cGy (gamma irradiation from a linear accelerator) prior to their further conditioning for the study.

The first experiment (referred to as experiment 1) used a total of 20 female mice (*n* = 20) in three groups. The macrophage-depletion group (*n* = 5) received intravenous clodronate containing liposome injection just after their TBI, followed by the intravenous injection of 30 × 10^6^ human peripheral blood mononuclear cells (huPBMCs) the day after. The nonmacrophage-depleted group (*n* = 10) only received huPBMCs the day after TBI. The mice in the control group (*n* = 5) were irradiated but did not receive huPBMCs nor clodronate.

The second experiment (referred to as experiment 2) used 16 male mice (*n* = 16) in two groups. The macrophage-depleted group (*n* = 8) received clodronate-containing liposomes after TBI, followed by intravenous injection of huPBMCs from 2 different donors the following day (containing at least 10 × 10^6^ T-cells: 6 × 10^6^ huPBMCs for donor A and 10 × 10^6^ huPBMCs for donor B), and the nondepleted group (*n* = 8) only received these huPBMCs the day following TBI.

Mice were monitored for symptoms of GvHD as described previously [28]. Mice were sacrificed by cervical dislocation after 70 days or when losing more than 20% of their original body weight or when they experienced severe symptoms, wound infection of signs of bleeding caused by the given injections.

#### 2.1.2. Preparation of Clodronate-Containing Liposomes

Clodronate-containing liposomes were prepared as described by van Rijn et al. [26]. Briefly, a mixture of egg-phosphatidylcholine, egg-phosphatidylglycerol (both from Lipoid, Ludwigshafen, Germany), and cholesterol (Sigma, St. Louis, MO) was dissolved in ethanol in a molar ratio of 10 : 1 : 1.5 and evaporated to dryness by rotation under reduced pressure. The lipid film was hydrated in an aqueous solution containing clodronate (Cl2MDP, concentration 60 mg/mL, Bonefos; Schering, Weesp, Netherlands). The unencapsulated clodronate was removed by repeated washing using PBS pH 7.4 and ultracentrifugation (Beckman Optima LE-80 K; Palo Alto, CA) at 200,000 g for 30 min. After washing, the pellet was resuspended in PBS at a concentration of 90 mM phospholipids. The final concentration of clodronate was determined using spectrophotometry at a wavelength of 238 nm after extraction and binding to Cu^+^ and appeared to be within a range of 2-2.5 mg/mL. Mice prone for depletion of macrophages received 0.2 mL of the liposome-suspension intravenously one day before injection of huPBMCs.

### 2.2. B-Cell Depletion Model

B-cell depletion was studied, using 30 female RAG2^−/−^*γ*c^−/−^ mice, separated in 4 groups. All mice received TBI one day prior to further conditioning as described below. Injection of Rituximab and/or human cells was performed intravenously. One group of mice (*n* = 10) was injected with 50 *μ*g Rituximab followed by 7.5-15 × 10^6^ huPBMCs. The second group (*n* = 10) was injected with huPBMCs only, the third group (*n* = 5) with 50 *μ*g Rituximab only, and the fourth group (*n* = 5) received TBI only.

Mice were monitored for development of symptoms of GvHD as described previously [28] and sacrificed by cervical dislocation either after 70 days or when either losing more than 20% of their original body weight, having severe symptoms, wound infection, or bleeding because of the given injections or when they experienced side effects of the medication. FACS analysis with a FACSCalibur (Becton Dickinson) was performed to evaluate the fraction of human cells in the murine peripheral blood, using human CD45 (huCD45), mouse CD45, huCD3, and huCD19 (Becton Dickinson), as described by van Rijn et al. [26].

### 2.3. Preparation and Transplantation of huPBMCs (Both Models)

HuPBMCs were prepared as described previously [26]. Briefly, fresh buffy coats were obtained from healthy human volunteers at the Blood Bank of the University Medical Centre Utrecht, and huPBMCs were isolated using Ficoll Hypaque (Pharmacia, Uppsala, Sweden) density centrifugation. Cells were washed twice in a phosphate-buffered saline (PBS) and resuspended in PBS/0.1% human serum albumin (HSA). Fresh cell suspensions of 0.2 mL (containing huPBMCs in concentration as stated above and using a different human donor in each experiment) were injected intravenously in the tail vein, without prior ex vivo stimulation.

### 2.4. Histology and Immunohistochemistry (Both Models)

#### 2.4.1. In General

Autopsy was performed on all mice in both the macrophage-depletion model and the B-cell depletion model. The spleen, lungs, liver, ileum, colon, skin (from cervical region), and femur were collected for further analysis. Each organ was split (femur split as being right sided and left sided); one part was frozen and stored in liquid nitrogen and another part was fixed in PBS-buffered formaldehyde (4%). The femur was decalcified with EDTA. The formalin-fixed organs were embedded in paraffin, and tissue sections were cut at 4 *μ*m using coated slides, for staining with haematoxylin-eosin (H&E) for histology and several primary antibodies for immunohistochemistry (Table 1) as described previously [28]. In our second macrophage-depletion study, tissue microarray (TMA) blocks were made.

Immunohistochemical staining was performed on slides from FFPE-blocks in a Bond-maxTM automated immunostainer (Leica Microsystems, United Kingdom) after pretreating automatically with either citrate, using the Bond Epitope Retrieval Solution 1 (AR9961), or EDTA, using the Bond Epitope Retrieval Solution 2 (AR9640) with washing steps between each reagent using 1× Bond Wash Solution. The staining was identified by a polymer-based detection system with Diaminobenzidine (DAB) using the Bond Polymer Refine Detection kit (DS9800). The sections were counterstained with haematoxylin.

For human FoxP3 staining, slides were deparaffinized, washed, and blocked with endogenous peroxidase blocker. Antigen retrieval was performed using citrate pretreatment; slides were then washed and incubated with diluted anti-human FoxP3 antibody for 1 hr. After rinsing, slides were incubated for 30 min with rabbit-anti-rat-HRP (DAKO, Glostrup, Denmark), diluted at 1 : 250, and incubated with PowerVision Goat-anti-rabbit IgG HRP (Immunologic, Klinipath, Netherlands), followed by DAB staining for 10 min after washing with PBS. Slides were counterstained with haematoxylin.

Mouse-CD68 staining was performed on frozen tissue sections cut at 8 *μ*m and stained as described previously [28] using a combination of rabbit-anti-mouse antibody-HRP (DAKO) and 10% mouse serum (DAKO) in a dilution of 1 : 100. Staining was identified using DAB and counterstaining with haematoxylin.

Cross-reactivity of all monoclonal and polyclonal antibodies used (against human antigens) was tested using both a positive control (human tonsil) and a double negative control. These negative controls consisted of a control staining using BALB/c tissue and of murine tissue from RAG2^−/−^*γ*c^−/−^ mice without huPBMCs. In these RAG2^−/−^*γ*c^−/−^ mice without huPBMCs, a control was run with primary antibody and a control staining using murine tissue with human cells without the primary antibody to rule out both false positive staining due to cross-reactivity of the other reagents used and false negative staining for the primary antibody. Cross-reactivity of antimurine CD68 was tested in human tonsil tissue.

Slides were assessed for presence, severity, and location of both fibrosis and cell infiltrate, including the huPBMCs and murine macrophages by two experienced pathologists and an experienced molecular biologist using light microscopy, using our previously described method [28].

#### 2.4.2. Macrophage-Depletion Model

In these experiments, the spleen, lungs, liver, ileum, colon, skin, and femur were isolated from each mouse. From the first experiment, only 9 mice could be used for histological analysis of all these organs (a total of *n* = 9 per organ; *n* = 6 for nondepletion and *n* = 3 for macrophage depletion). From the second experiment, 16 mice were available for both histological and cytokine analysis (adding up to *n* = 16 per organ; *n* = 8 for nonmacrophage depletion and *n* = 8 for macrophage depletion).

#### 2.4.3. B-Cell Depletion Model

The spleen, lungs, liver, ileum, colon, skin, and femur were isolated, adding up to a total of *n* = 28 per organ available for complete analysis, separated in 4 groups (*n* = 10 for Rituximab with huPBMCs, *n* = 9 for huPBMCs only, *n* = 3 for Rituximab only, and *n* = 5 for neither huPBMCs nor Rituximab).

### 2.5. Cytokine Profile Analysis

The cytokine gene expression profile was analyzed by quantitative polymerase chain reaction (Q-PCR) to measure the amount of human and murine messenger RNA (mRNA) in the tissues.

For macrophage depletion, RNA was isolated from all mice for the spleen, liver, lung, skin, and bone marrow. For B-cell depletion analysis, the spleen, liver, lung, skin, colon, ileum, and femur were isolated and cytokine analysis was performed for several murine and human cytokines as described previously [13].

RNA was isolated using the RNeasy Mini Kit (Qiagen, Venlo, Netherlands), according to the manufacturer's instructions. Of each sample, 3 *μ*g RNA was incubated with 1 *μ*L of both oligoDT (Promega, Leiden, Netherlands) and Random Primers (Promega) for 5 min at 70°C. Finally, cDNA was synthesized by adding a mixture of first strand buffer 5× (Invitrogen, Breda, Netherlands), 0.1 M DTT, 10 mM dNTPs (Invitrogen), and incubation for 1 hr at 42°C. The cDNA was stored upon use at -20°C.

For all cytokines and other gene-targets tested, the human and mouse specificity was determined by testing the primer-probe combination against mRNA isolated from normal human and murine tissues (RAG2^−/−^*γ*c^−/−^ and BALB/c mice). Only species-specific predeveloped TaqMan Assays were used (TaqMan Gene Expression Assay: Applied Biosystems, Foster City, CA). The Q-PCR reactions were run on either a LightCycler 480 real-time PCR system (Roche Diagnostics, Lewes, UK) or a ViiA 7 Real-Time PCR system (Thermo Fisher Scientific, Landsmeer, Netherlands). Each sample was run in duplicate, and a negative control was used to exclude contamination. GAPDH was used as reference gene. The relative mRNA quantity was determined using the E-method of the LightCycler 480 software (Roche) and the ViiA 7 software (Thermo Fisher Scientific). In this method, a correction for PCR efficiency is embedded in the calculation of the relative quantity of gene expression (RQ value).

All samples were analyzed for RNA levels of human TGF-*β*IFN-*γ*, and CXCR4, as well as fibrosis-related factors like FGF2, PDGF, EGF, BMP4, and CTGF and multiple cell-specific factors (FoxP3, GATA3, Tbet, PAI, RORC, CD4, CD8, CD20, and CD68) in order to further specify the involved cells. Samples were also analyzed for murine markers TGF-*β*, CTGF, BMP4, CD68, and INF-*γ*. An explanatory list of the abbreviated cytokines and markers is included in Table 2.

## 3. Results

### 3.1. Clinical Symptoms

#### 3.1.1. Macrophage-Depletion Model

**Figure 1:**
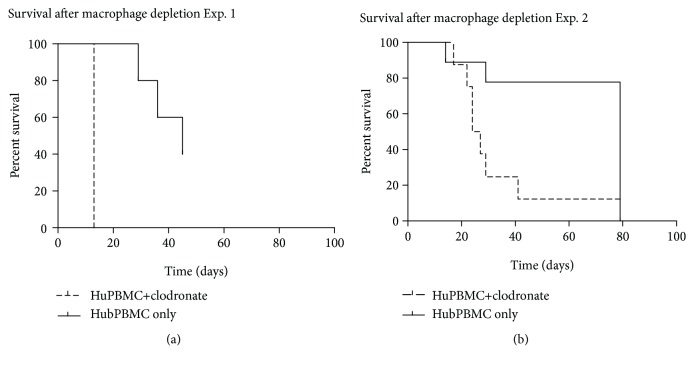
Survival of RAG2^−/−^*γ*c^−/−^ mice after introducing HuPBMCs with or without clodronate-driven depletion of macrophages. (a) Results from experiment 1. Mean survival with macrophage depletion (dotted line) was 13 days (SD 0), mean survival without macrophage depletion (continuous line) 36.67 days (SD 7.174) after intravenous injection with huPBMCs. (b) Results from experiment 2. Mean survival 32.88 days (SD 19.0) with macrophage depletion (dotted line) and mean survival 64.63 days (SD 26.9) without macrophage depletion (continuous line, pooled data from both donors after huPBMC injection).

Both macrophage-depletion experiments showed a significant difference in survival, in which survival of mice depleted with macrophages was impaired (*p* = 0.0005 in experiment 1 and *p* = 0.0099 in experiment 2). Kaplan-Meier curves were plotted to compare survival in both experiments (Figure 1). In both experiments, the mice showed severe weight loss, ruffled fur, reduced mobility, erythema of the skin, and in a few splenomegaly (mostly in mice without macrophage depletion). Macrophage depletion was considered effective as a significant difference between clodronate-treated mice, and nontreated mice was determined that could not be explained otherwise. qPCR analysis for macrophages (CD68) was performed at time of death related to the initiated Graft-versus-Host response (see below), with mean survival of mice treated with clodronate being 13 days in our first experiment and 25.5 days in our second experiment (with mean survival after codronate treatment was 22 days for donor A and 27 days for donor B).

#### 3.1.2. B-Cell Depletion Model

**Figure 2:**
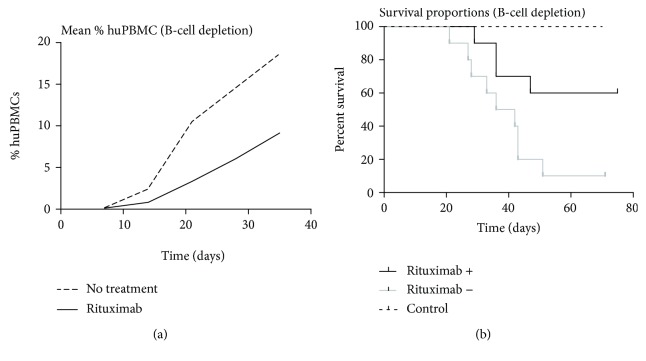
Survival and comparison of fraction reconstituted huPBMCs in B-cell depletion. (a) The difference in percentage of huPBMCs in mice either treated with Rituximab to induce B-cell depletion or not treated with Rituximab, and (b) a comparison of the survival portions between the RAG2 ^−/−^*γ*c^−/−^ mice with and without Rituximab treatment versus control mice (*n* = 10), consisting of 5 RAG2 ^−/−^*γ*c^−/−^ mice with Rituximab but no huPBMCs and 5 RAG2 ^−/−^*γ*c^−/−^ control mice (with no Rituximab nor huPBMCs).

B-cell depletion was considered successful as histologically; numbers of B-cells were significantly reduced compared to mice not treated with Rituximab (see below). The huPBMC-injected groups showed a significant difference in survival (*p* = 0.02), with a beneficial effect on survival after Rituximab treatment. Survival was also significantly related to the development of GvHD symptoms, and developing symptoms were significantly related to the amount of huPBMCs reconstituting the mice (*p* < 0001). Symptoms of affected mice again included severe weight loss, ruffled fur, reduced mobility, erythema, and in some cases splenomegaly. The number of circulating huPBMCs appeared to be lower after Rituximab treatment (Figure 2), but this did not reach statistical significance (*p* = 0.2046).

### 3.2. Histomorphological Infiltrate Analysis

#### 3.2.1. Macrophage-Depletion Model

Infiltration patterns of cells and fibrosis in both experiments followed the known pattern for this xenogeneic model (see reference (28) about the description of this model). In our first experiment, macrophage depletion showed significantly increased B-infiltration in the liver (based on CD79a), lung, and femur (both based on CD20). In addition, a significant increase was found on the number of plasma cells in the bone marrow (CD138) (Table 3).

The overall analysis of the second experiment showed significantly increased fibrosis in the femur, an increased number of T-cells in the liver, lung, skin, colon, and femur, increased numbers of B-cells in the spleen, liver, and lung, and a variable increase or decrease in the number of plasma cells in the spleen, liver, and lung (Table 3), though when comparing the different donors used, this significance did not apply for all donors at all times.

Macrophage depletion did not show any effect on the number of Tregs in the morphological evaluation.

#### 3.2.2. B-Cell Depletion Model

Infiltration patterns of cells and fibrosis followed the known pattern for this xenogeneic model from our previous description [28]. Early depletion of B-cells showed a significant decrease in the numbers of B-cells and plasma cells in the spleen and liver (in which the Rituximab treated huPBMC-injected group did not show any B-cells nor plasma cells at all), as well as a significant decrease of fibrosis in the liver (Table 4). When comparing only the huPBMC-injected groups, a significant decrease was shown for the presence of B-cells and plasma cells in the lung (*p* < 0.01 and *p* < 0.05, respectively) in which again no B-cells or plasma cells were detected in the Rituximab treated huPBMC-injected mice, as well as a significant decrease of the presence of T-cells in the gut (*p* < 0.05).

### 3.3. Cytokine Profile

#### 3.3.1. Macrophage-Depletion Model

**Figure 3:**
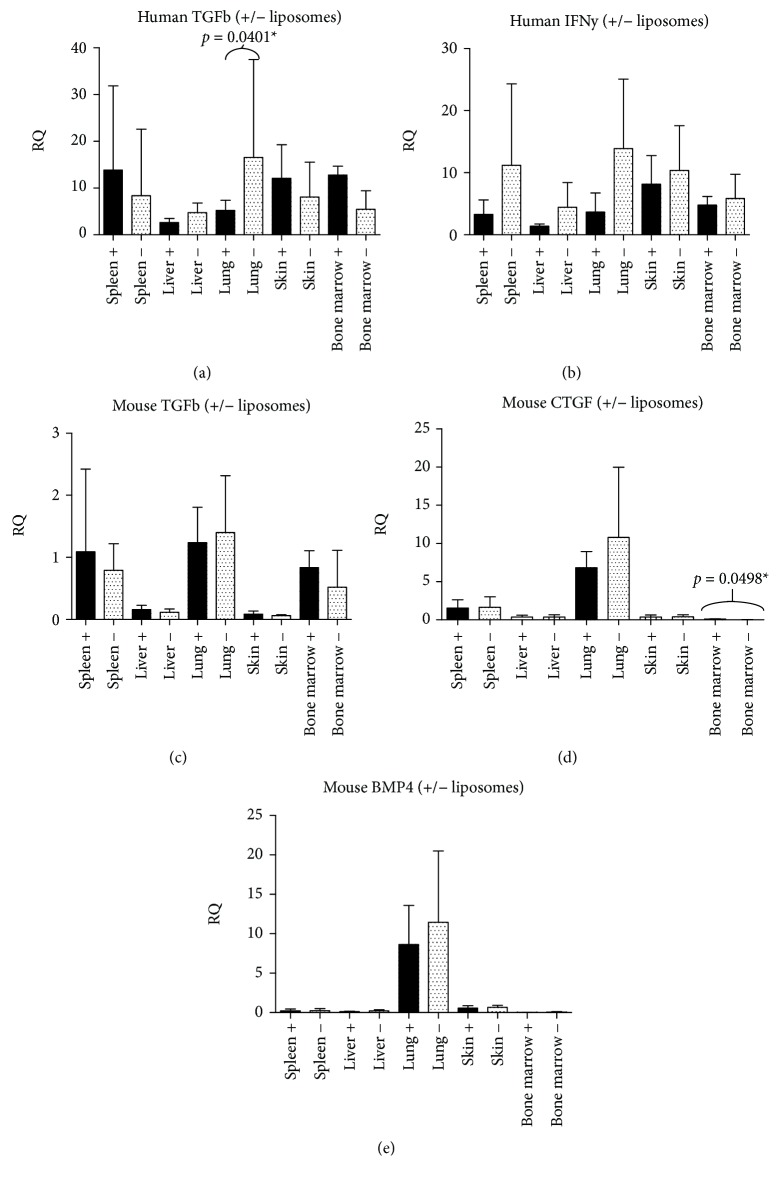
Expression of mRNA for fibrogenic proteins in macrophage depletion. The RQ values of mRNA expression are shown from macrophage-depletion experiment 2, for RNA for human TGF-*β*, human IFN-*γ*, and for murine proteins TGF-*β*, CTGF, and BMP4 in the spleen, liver, lung, skin, and bone marrow of RAG2 ^−/−^*γ*c^−/−^ mice injected with huPBMCs with macrophage depletion due to clodronate-containing liposomes (indicated as +) and RAG2 ^−/−^*γ*c^−/−^ mice injected with huPBMCs without this depletion (indicated as -). No murine INF-*γ* was detected.

The RNA quality from the first experiment was not sufficient for cytokine analysis. Cytokine analysis performed in the second experiment showed that human macrophages (huCD68) were not present (cycle thresholds were above 30 in all samples). No significant difference was detected for murine macrophages (mouse CD68); more specifically, no differences were detected in the spleen (*p* = 0.2284) nor the liver (*p* = 0.1304). Minor significant differences were detected for other cytokines (Figure 3). Murine IFN-*γ* was not detected, but murine TGF-*β*, CTGF, and BMP4 were present in several organs as well as human TGF-*β* and IFN-*γ* and RNA specific for Tregs (FOXP3), Th1 cells (Tbet), Th2 cells (GATA3), and Th17 cells (RORC).

**Figure 4:**
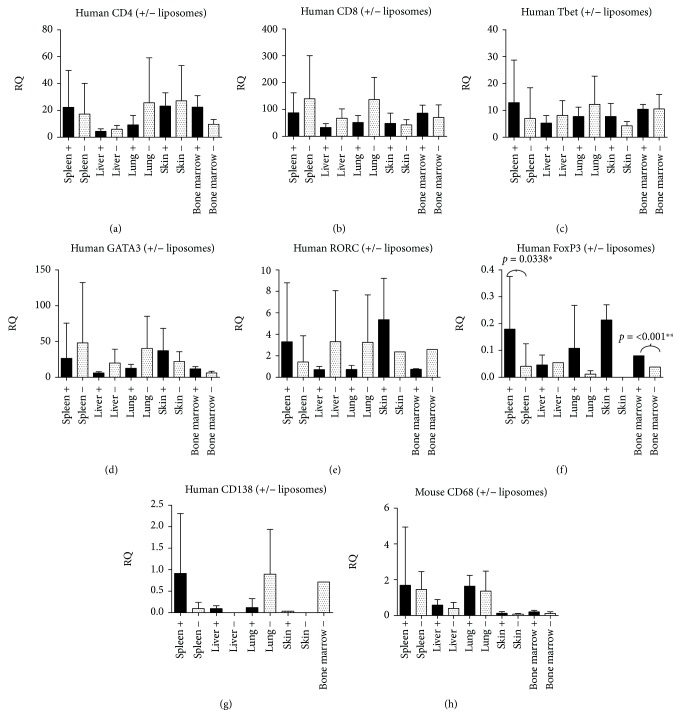
Expression of mRNA for specific cell marker mRNA in macrophage depletion. RQ values are shown from macrophage-depletion experiment 2; RAG2 ^−/−^*γ*c^−/−^ mice injected with huPBMCs with macrophage depletion due to clodronate-containing liposomes (indicated as +) and RAG2 ^−/−^*γ*c^−/−^ mice injected with huPBMCs without this depletion (indicated as -), for different human cell types, including FoxP3 (Tregs), GATA3 (Th2 cells), Tbet (Th1 cells), RORC (Th17 cells), CD4 (T helper cells), CD8 (cytotoxic T-cells), and CD138 (plasma cells) as well as for murine CD68 (murine macrophages). Human CD68 showed cycle threshold values above 30 only and human macrophages were therefore considered as virtually not present.

A decrease for human TGF-*β* was detected in the lungs and an increase in mouse CTGF in the bone marrow. Although human IFN-*γ* levels in several organs seemed to be lower after macrophage depletion, this effect was not significant. Comparing mRNA levels for specific cell markers (Figure 4) showed that in contrast to the histological analysis, a significant increase in human FoxP3 was found in the spleen (*p* = 0.0038) as well as in the bone marrow (*p* < 0.001). In the experiments with macrophage depletion, the levels of FoxP3 positive cells were usually higher in these organs (Figure 4).

#### 3.3.2. B-Cell Depletion Model

**Figure 5:**
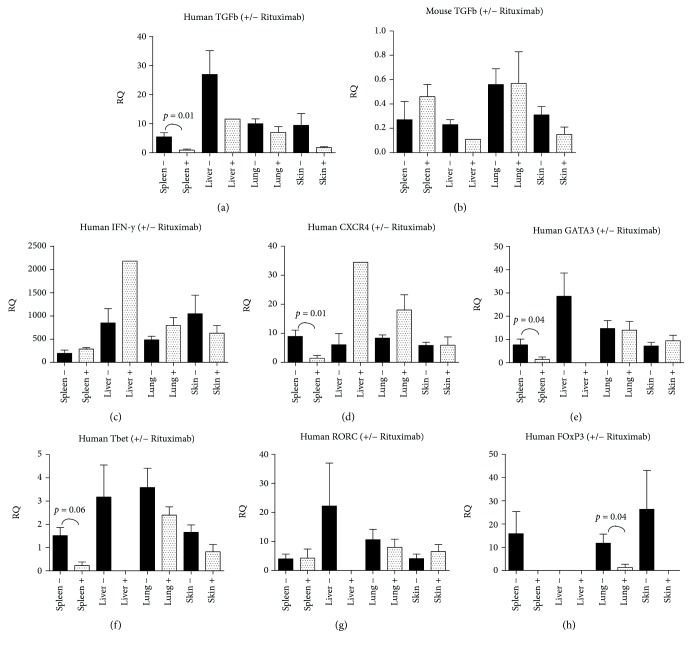
Expression of mRNA for fibrogenic proteins and specific cell marker mRNA in early B-cell depletion. RQ values are shown from the early B-cell depletion experiment; RAG2 ^−/−^*γ*c^−/−^ mice injected with huPBMCs with Rituximab induced B-cell depletion (indicated as +) and RAG2 ^−/−^*γ*c^−/−^ mice injected with huPBMCs without this depletion (indicated as -), for the most important observations, concerning human and murine TGF-*β*, human IFN-*γ*, and different human cell types, including FoxP3 (Tregs), GATA3 (Th2 cells), and Tbet (Th1 cells). All other results were either not significant (CD4, CD8, murine CD68, murine CTGF, murine BMP4), had cycle threshold (Ct) values > 35 (CD20 and human CD68) or were not detectable at all (human factors FGF2, PDGF, EGF, BMP4 and CTGF).

Similar cytokine analysis in our B-cell depletion model (Figure 5) confirmed the presence of murine macrophages and murine markers CTGF, BMP4, and TGF-*β*. No murine IFN-*γ* was detected. Human markers FG2, PDGF, EGF, BMP4, and CTGF were not detected, but variable RQ values for human TGF-*β*, IFN-*γ*, FoxP3, CXCR4, GATA3, Tbet, PAI, and RORC were detected.

The results showed a significant difference for human TGF-*β* in the spleen (*p* = 0.01), levels being higher in mice with Rituximab treatment. Human TGF-*β* levels in the liver also seem to be higher, but as only one mouse could be evaluated with Rituximab treatment, the significance of this difference could not be established. A similar problem occurred for FoxP3, CXCR4, GATA3, Tbet, and RORC (number of adequate samples for evaluation were too low), although the spleen and the lung both show a significantly higher level of FoxP3 in mice without treatment (*p* = 0.04) and CXCR4, GATA3, and Tbet also were significantly increased in the spleen (*p* = 0.01, *p* = 0.04, and *p* = 0.006) in the nontreated group. We did not find significant differences in the number of CD4+ or CD8+ cells.

## 4. Discussion

Our study evaluated the effect on survival, histomorphological changes, and cytokine assays in macrophage depletion as well as early B-cell depletion in a xenogeneic mouse model for GvHD. Although our experiments may show a substantial difference in the number of and gender of mice used (as the number of mice treated was subject to availability of cells and clodronate as well as the availability of an adequate number of mice of similar gender and age), we consider the results of our experiments sufficient to address the issue of possible significant effects of modifications in xenogeneic mouse models for GvHD when using macrophage- or B-cell depletion protocols, even when only single dose treatment of either clodronate-containing liposomes of Rituximab has been used.

With depletion of macrophages, survival was significantly impaired. Histologically, the number of B-cells and occasionally plasma cells in the liver, lung, and femur was altered. The effect on the histological presence of fibrosis was unremarkable and only visible in the femur. The molecular analysis showed a decrease in human TGF-beta in the lungs and an increase in murine CTGF in the bone marrow after our clodronate depletion protocol. We did not find significant changes in murine CD68 in our qPCR analysis, though we believe that this reflects the known regeneration of murine macrophages after a single dose treatment with clodronate-containing liposomes as reported in previous literature [22, 24, 25] as our qPCR analysis was performed at least 13 days posttreatment and regeneration starts within 5 days in the liver and within a week in the spleen.

With our early B-cell depletion experiment, the clinical effect was precisely opposite to macrophage depletion. Survival was clearly improved, even though the number of circulating huPBMCs did not change significantly. In addition to histological confirmation of B-cell depletion, the histomorphological analysis only showed a change in liver fibrosis and no significant alterations in infiltrating T-cells or Tregs. The cytokine analysis in our Rituximab experiment showed several interesting observations. There were decreased human TGF-beta levels in the spleen, as well as decreased levels for CXCR4, GATA3 (Th1 cells), and Tbet (Th2 cells). The observed reduction in CXCR4 in the spleen suggests that lymphocytes are less drawn to the spleen after early Rituximab treatment. Our results may suggest an increase in CXCR4 in the liver, lungs, and skin, indicating that perhaps these organs become more attractive to lymphocytes after Rituximab treatment. However, the qPCR results in these organs were not significant. The decrease in GATA3 and Tbet without changes in the number of CD4+ and CD8+ cells (neither histologically nor in qPCR) remains difficult to explain. One might expect an increase in Th17 cells (with an increase in RORC in qPCR analysis) to compensate and explain our histological data, but our qPCR data could not confirm this (no significant changes in RORC were seen). It will be interesting to look into this in more detail in future experiments.

Our clinical macrophage-depletion results are in line with the clinical effect as described in literature, including the lack of human macrophages in general after introducing human cell mixtures [26, 29]. The obtained reaction develops faster, and symptoms are more severe after macrophage depletion. Our results show, however, that the number of infiltrating B-cells and occasionally plasma cells might significantly increase after a macrophage-depletion protocol. To our knowledge, this is a new observation that has not been described before.

Our early B cell depletion experiment also follows the known pattern of affected survival, but despite the clear clinical effect, the morphology in the evaluated murine organs only showed a difference in liver fibrosis between mice treated and not treated with Rituximab.

The observation of only alterations in liver fibrosis and the effect on TGF-beta does not contradict the encountered profound clinical effect. Liver fibrosis causes fatigue, nausea, and weight loss, and in our model, we can only conclude that the liver fibrosis is due to a xenogeneic GvHD.

However, as we could only establish a difference in liver disease, the clinical improvement therefore does not imply that the effects account for all clinical features of GvHD (e.g., cutaneous involvement). Also, in human, other therapeutic agents or comorbidities may have an effect on liver function as well and with that become a confounder in the evaluation of, e.g., Rituximab on clinical performance. The increase in human TGF-*β* is in line with previous literature [6] and might be the cause for our liver fibrosis as murine CTGF and murine BMP4 did not show significant changes. TGF-*β* is known for its effects on inducing injury in hepatocytes [30, 31]. The levels of TGF-*β* in the liver could unfortunately not be evaluated for significance, as only a small number of mice treated with Rituximab had an adequate RNA sample to process. If this would follow the significance as seen in the spleen and lungs for this profibrogenic protein, this could be an explanation for the cause of fibrosis.

Our Q-PCR data also suggest an interplay between human TGF-*β* and murine tissue. This is in line with the suggestion by MacDonald et al. [32] that TGF-*β* is indeed derived from engrafting donor-cells, although they correlated this to donor macrophages and we only affected donor B-cells in this experiment. For donor macrophages, all cycle thresholds in our Q-PCR analysis were above 30, concluding that virtually, no donor macrophages were present. Only host macrophages could be detected. It is known that host macrophages (in their function as antigen-presenting cells) are related to the occurrence of GvHD [33]. Our macrophage-depletion experiments confirm the importance of host macrophages in GvHD. The observed reduced survival due to depletion of host macrophages in our experiments could possibly be related to the loss of Toll-like receptor 4 (TLR-4) fin macrophages, as described by Imado et al. [34]. However, urther research is suggested to unravel the possible interactivity between host, donor macrophages (if present), and (perhaps different activation stages of) donor B-cells in GvHD.

It would be very interesting to correlate our histological findings with clinical hepatic GvHD. So far, no literature was found on the effects of Rituximab in hepatic GvHD specifically. Nevertheless, for cutaneous GvHD, a correlation between TGF-*β* and the length of survival has already been shown, even though the results show that in humans, the relation probably only accounts for acute GvHD and lichenoid chronic GvHD [35]. It supports our findings of the importance of TGF-*β* in GvHD, although our study is showing a more sclerotic type of response.

With regard to Tregs, our B-cell depletion experiment showed two interesting features.

First, in our mice with less symptoms (after Rituximab treatment), the mRNA levels of FoxP3 were significantly lower compared to those with a more profound reaction, although literature suggests that higher levels of Tregs should correlate with increased survival. However, the low mRNA level of FoxP3 was not accompanied by a change in Treg infiltration histologically, suggesting that the mRNA level of FoxP3 is not always linear related to the actual number of Tregs. Either it reflects the difficulty of scoring less-frequent cells histologically or the FoxP3 protein might be upregulated within single Tregs without a significant increase in the number of Tregs. Our observation is consistent though, with the findings of Wu et al. [35] in their skin biopsies.

Second, when correlating our results with literature, the findings of lower mRNA levels for FoxP3 in the spleen and lung after Rituximab treatment suggests that our early B-cell depletion affected mostly inactivated B-cells [11]. Indeed, we introduced Rituximab almost immediately after the introduction of huPBMCs. We did not know the number of activated B-cells in our huPBMC mixture nor the number of CD20+CD5+ cells (an indicator for possible clinical response to treatment [7]), and therefore, these findings might well be related to each other.

In summary, we confirm known clinical effects of depleting macrophages or B-cells in a GvHD mouse model, but our experiments showed that histological and cytokine assay results can be significantly influenced by depletion of either of these cell lines as well. For both cell lines, the effect of depletion on histological changes can be unexpected despite significant effects on survival. Cytokine-analysis might however help explain some of the encountered contradictions and the interplay between donor and host cells. We, therefore, strongly continue to recommend researchers interested in GvHD to always perform a full triad analysis of clinical symptoms, histology, and cytokine profile (specified not only for peripheral blood but also for other involved organs in the reaction).

## Figures and Tables

**Table 1:** Antibodies used for immunohistochemical detection of human and murine cells after huPBMC injection in RAG2^−/−^*γ*c^−/−^ mice.

Primary antibody	Company	Animal	Mono/poly	Clone	Lot	Dilution	Pretreatment
Hu-CD2	*Novocastra, United Kingdom*	Mouse	Monoclonal	AB75	127119	1 : 160	EDTA
Hu-CD4	*Monosan Xtra*, *Netherlands*	Mouse	Monoclonal	4B12	247919	1 : 200	EDTA
Hu-CD8	*DakoCytomation*, *Denmark*	Mouse	Monoclonal	C8/144B	51182	1 : 200	Citrate
Hu-CD20	*DakoCytomation*, *Denmark*	Mouse	Monoclonal	L26	83	1 : 400	Citrate
Hu-CD79a	*DakoCytomation*, *Denmark*	Mouse	Monoclonal	JCB117	42791	1 : 200	Citrate
HuCD138	*Serotec*, *Germany*	Mouse	Monoclonal	B-B4	605	1 : 1000	Citrate
Hu-kappa	*DakoCytomation*, *Denmark*	Rabbit	Polyclonal	.	44399	1 : 10000	Citrate
Hu-lambda	*DakoCytomation*, *Denmark*	Rabbit	Polyclonal	.	120	1 : 20000	Citrate
Hu-plasmacell	*DakoCytomation*, *Denmark*	Mouse	Monoclonal	VS38c	38925	1 : 400	EDTA
HuCD68	*Novocastra*, *United Kingdom*	Mouse	Monoclonal	KP1	211708	1 : 800	Citrate
Hu-FoxP3	*eBioscience*, *United Kingdom*	Rat	Monoclonal	PCH101	E021753	1 : 200	Citrate
Mouse-CD68	*Hycult Biotech*, *Netherlands*	Rat	Monoclonal	FA-11	4817M21	1 : 50	None

**(a):** 

Abbreviations used in qPCR analysis regarding specific cytokine/protein/gene function		
TGF-*β*/TFGb	Transforming growth factor beta	Multifunctional cytokine known for its role in regulating inflammatory processes and involved in proliferation, activation, and chemotaxis of many immune cells
CTLA-4	Cytotoxic T-lymphocyte-associated antigen 4	Protein receptor involved in downregulation of immune responses, expressed on T cells
GAPDH	Glyceraldehyde-3-phosphate dehydrogenase	Protein coding gene, known as “housekeeping” gene
IFN-*γ*	Interferon gamma	Important regulating cytokine in inflammatory processes, with immunostimulatory and immunomodulating effects
CXCR4	Chemokine receptor type 4	Receptor for stromal derived factor 1 (chemotactic for lymphocytes and important for hematopoietic homing of stem cells)
FGF2	Fibroblast growth factor 2	Fibrosis-related factor with mitogenic effects on fibroblasts.
PDGF	Platelet derived growth factor	Fibrosis related factor with mitogenic effects on fibroblasts
EGF	Epidermal growth factor	Promotor of TGF-*β*-related fibrosis
BMP4	Bone morphogenetic protein 4	Proinflammatory gene with profibrotic potential
CTGF	Connective tissue growth factor	Profibrotic cytokine

**(b):** 

Abbreviations used in qPCR analysis regarding specific cell types	
FoxP3	Abbreviation for Forkhead protein 3, used as signature for regulatory T cells (Tregs)
GATA3	Transcription factor involved in T helper 2 (Th2) cell differentiation
Tbet	Transcription factor associated with T helper 1 (Th1) cell differentiation
RORC	Signature for T helper 17 (Th17) cells
CD4	T helper cell cluster
CD8	Cytotoxic T cell population
CD20	B cell population
CD68	Macrophage population

**Table 3:** Statistical analysis of histology and immunohistochemistry data in macrophage depletion. Mean survival with Standard Deviation (SD) *p* values for histological and immunohistochemical scoring in macrophage-depletion experiments 1 and 2, each experiment comparing mice injected with huPBMCs with macrophage depletion by clodronate-containing liposome injection (liposomes+) and mice injected with huPBMCs without clodronate-containing liposome injection (liposomes-). The encountered effect related to liposomes injected is indicated with arrows indicating an associated increase or decrease of the mean histological infiltration scores.

				Fibrosis			T-cells			B-cells (CD79a)			B-cells (CD20)			Plasma cells			Tregs		
			N (+/- liposomes)	Effect of liposomes	*p* value	Significant	Effect of liposomes	*p* value	Significant	Effect of liposomes	*p* value	Significant	Effect of liposomes	*p* value	Significant	Effect of liposomes	*p* value	Significant	Effect of liposomes	*p* value	Significant
Spleen	Exp. 1	Single donor	10(3/7)	↓	0.22	No	↓	0.22	No	↓	0.11	No	↑	0.11	No	↓	0.68	No	↑	0.21	No
	Exp. 2	Pooled data (donor A+B)	16 (8/8)	↑	0.28	No	↑	0.16	No	↑	<0.0001	Yes	↑	<0.0001	Yes	↑	0.0008	Yes		#	#
		*Donor A only*	*9 (5/4)*	↓	*0.0035*	*Yes*	↓	*0.1131*	*No*	↑	*<0.0001*	*Yes*	↑	*0.0314*	*Yes*	↑	*<0.0001*	*Yes*		#	#
		*Donor B only*	*7 (3/4)*	↑	*0.5514*	*No*	↑	*0.0321*	*Yes*	↑	*0.0013*	*Yes*	↑	*0.5857*	*No*	↑	*0.2112*	*No*		#	#

Liver	Exp. 1	Single donor	10(3/7)	↓	0.06	No	↓	0.11	No	↓	0.04	Yes	↑	0.01	Yes	↓	0.48	No	↑	0.17	No
	Exp. 2	Pooled data (donor A+B)	16 (8/8)	↑	0.1	No^∗^	↑	0.0006	Yes	↑	0.002	Yes	↑	0.002	Yes	↑	0.0003	Yes		#	#
		*Donor A only*	*9 (5/4)*	↑	*0.0031*	*Yes* ^∗^	↑	*0.0001*	*Yes*	↑	*0.0005*	*Yes*	↑	*0.0431*	*Yes*	↑	*0.006*	*Yes*		#	#
		*Donor B only*	*7 (3/4)*	↓	*0.6447*	*No*	↑	*0.3743*	*No*	↓	*0.4178*	*No*	↑	*0.2367*	*No*	↑	*0.0101*	*Yes*		#	#

Lung	Exp. 1	Single donor	10(3/7)	↓	0.21	No	↓	0.31	No	↓	0.06	No	↑	0.04	Yes	↓	0.06	No	↑	0.1	No
	Exp. 2	Pooled data (donor A+B)	16 (8/8)	↓	0.25	No^∗^	↑	0.01	Yes	↑	0.028	Yes	↑	0.028	Yes	↑	0.0004	Yes		#	#
		*Donor A only*	*9 (5/4)*	≈	*0.393*	*No* ^∗^	↑	*0.05*	*Yes*	↑	*0.4231*	*No*	↑	*0.1094*	*No*	↑	*0.0202*	*Yes*		#	#
		*Donor B only*	*7 (3/4)*	↓	*0.5206*	*No* ^∗^	↑	*0.69*	*No*	↑	*0.0033*	*No*	↑	*0.8255*	*No*	↑	*0.0007*	*Yes*		#	#

Skin	Exp. 1	Single donor	10(3/7)	≈	1	No	↓	0.27	No	↓	0.11^∗∗^	No^∗∗^		#	#	↓	0.22	No	≈	1.00	No
	Exp. 2	Pooled data (donor A+B)	16 (8/8)	≈	0.35	No	↑	0.0044	Yes	↑	0.0237	Yes^∗^	↑	0.0237	Yes^∗^	↑	0.548	No		#	#
		*Donor A only*	*9 (5/4)*	↑	*0.23*	*No* ^∗^	↑	*0.036*	*Yes*	↑	*0.013*	*Yes* ^∗^		#	#	↓	*0.3927*	*No*		#	#
		*Donor B only*	*7 (3/4)*	≈	*0.71*	*No*	↑	*0.1097*	*No*		#	#		#	#		#	#		#	#

Colon	Exp. 1	Single donor	10(3/7)		#	#	≈	0.89	No		#	#		#	#		#	#	↑	0.72	No
	Exp. 2	Pooled data (donor A+B)	16 (8/8)	↑	0.792	No^∗^	↑	0.0137	Yes	↑	0.88	No		#	#	≈	0.486	No		#	#
		*Donor A only*	*9 (5/4)*	≈	*0.9438*	*No* ^∗^	↑	*0.018*	*Yes*	↑	*0.5669*	*No*		#	#	↑	*0.3342*	*No*		#	#
		*Donor B only*	*7 (3/4)*	↑	*0.7439*	*No* ^∗^	↑	*0.5864*	*No*	↓	*0.4178*	*No*		#	#	↓	*0.4178*	*No*		#	#

Femur	Exp. 1	Single donor	10(3/7)	↑	0.68	No	↑	0.59	No	↑	0.89	No	↑	0.01	Yes	↑	0.04	Yes	↑	0.46	N o
	Exp. 2	Pooled data (donor A+B)	16 (8/8)	↑	0.0005	Yes	↑	0.0001	Yes	↑	0.0534	No	↑	0.0534	No	↑	0.253	No^∗^		#	#
		*Donor A only*	*9 (5/4)*	↑	*0.0075*	*Yes*	↑	*0.0012*	*Yes*	↑	*0.0057*	*Yes*	↑	*0.1074*	*No*	↑	*0.553*	*No* ^∗^		#	#
		*Donor B only*	*7 (3/4)*	↑	*0.076*	*No*	↑	*0.1251*	*No*	↑	*1.00*	*No*	↑	*!*	*!*	↑	*0.3165*	*No*		#	#

#: no cells/fibrosis detected, ^∗^significant difference between scoring of the analysts, ^∗∗^morphologically plasma cells with CD79a staining, !: number of available data not sufficient for analysis, ↑: mean score liposomes > mean score without liposomes, ↓: mean score liposomes < mean score without liposomes, ≈: mean scores almost equal.

**Table 4:** Statistical analysis of histology and immunohistochemistry data in B-cell depletion. Indicating the observed effect (increase or decrease of the encountered cell numbers) associated with the use of Rituximab in the huPBMC-injected groups, with the *p* values for histological and immunohistochemical scoring in mice injected with huPBMCs with and without Rituximab treatment and the control group without huPBMC injection.

	Fibrosis			T-cells			B-cells			Plasma cells			Tregs		
	Effect of Rituximab	*p* value	Significant	Effect of Rituximab	*p* value	Significant	Effect of Rituximab	*p* value	Significant	Effect of Rituximab	*p* value	Significant	Effect of Rituximab	*p* value	Significant
Spleen	↑	0.15	No	≈	0.92	No	↓	0.03	Yes	↓	0.03	Yes	↓	0.80	No
Liver	↓	0.05	Yes	↓	0.20	No	↓	0.03	Yes	↓	0.05	Yes	≈	0.92	No
Lung	↓	0.82	No	≈	1.00	No	↓	0.08^∗^	No^∗^	↓	0.14^∗∗^	No^∗∗^	↓	0.45	No

^∗^Overall effect, effect in huPBMC-injected groups is significant (*p* < 0.01) lacking B-cells in the huPBMC-injected group treated with Rituximab, ^∗∗^overall effect, effect in the huPBMC-injected group is significant (*p* < 0.05) lacking plasma cells in the huPBMC-injected group treated with Rituximab, ↑: mean score human cells Rituximab-treated group > mean score human cells without Rituximab treatment, ↓: mean score human cells Rituximab-treated group < mean score human cells without Rituximab treatment, ≈ mean score human cells in the Rituximab treated and nontreated group almost equal.

## Data Availability

The data used to support the findings of this study are available from the corresponding author upon reasonable request.
